# Application of the Uniform Data Set version 3 tele-adapted test battery (T-cog) for remote cognitive assessment preoperatively in older adults

**DOI:** 10.3389/fnagi.2024.1535830

**Published:** 2025-01-17

**Authors:** Mika M. Rockholt, Rachel R. Wu, Elaine Zhu, Raven Perez, Hamleini Martinez, Jessica J. Hui, Ekow B. Commeh, Romario B. Denoon, Gabrielle Bruno, Braden V. Saba, Daniel Waren, Courtney O'Brien, Vinay K. Aggarwal, Joshua C. Rozell, David Furgiuele, William Macaulay, Ran Schwarzkopf, Evan T. Schulze, Ricardo S. Osorio, Lisa V. Doan, Jing Wang

**Affiliations:** ^1^Department of Anesthesiology, Perioperative Care, Pain Medicine, New York University Grossman School of Medicine, New York, NY, United States; ^2^Interdisciplinary Pain Research Program, New York University Grossman School of Medicine, New York, NY, United States; ^3^Department of Orthopedic Surgery, New York University Grossman School of Medicine, New York, NY, United States; ^4^Department of Psychiatry, New York University Grossman School of Medicine, New York, NY, United States; ^5^Department of Neurology, New York University Grossman School of Medicine, New York, NY, United States; ^6^Department of Neuroscience and Physiology, New York University Grossman School of Medicine, New York, NY, United States

**Keywords:** neurocognitive dysfunction, perioperative cognitive dysfunction, remote cognitive assessment, screening, feasibility, neurocognitive disorders

## Abstract

**Introduction:**

Older adults undergoing surgery are at risk of postoperative neurocognitive disorders, prompting the need for preoperative cognitive screening in this population. Traditionally, cognitive screening has been conducted in-person using brief assessment tools such as the Montreal Cognitive Assessment (MoCA) or the Mini-Mental State Examination (MMSE). More comprehensive test batteries, such as the Uniform Data Set (UDS) Neuropsychological Battery, and its remote testing version, the Uniform Data Set version 3 tele-adapted test battery (UDS v3.0 T-cog), have been developed to assess cognitive decline in normal aging and disease conditions, but have not been applied in the perioperative setting.

**Methods:**

We assessed the feasibility of using this remote UDS v3.0 T-cog battery for preoperative cognitive assessment in 81 older adults 65+ scheduled for lower extremity joint replacement surgery.

**Results:**

Our results indicate that the UDS v3.0 T-cog achieves 99% completion rates and demonstrates high patient satisfaction. Further, we found 28% of subjects were cognitively impaired in this patient cohort.

**Discussion:**

These findings suggest that the UDS v3.0 T-cog is a feasible tool for assessing cognitive function in the older adult perioperative population. To our knowledge, this is the first study to apply this comprehensive remote test battery in the preoperative setting.

## 1 Introduction

The United States is experiencing a demographic shift, with the number of adults aged 65 years or older projected to grow from ~57 million in 2022 to 75 million by 2035, exceeding 20% of the total population.^1^ Older adults undergo 30% of all surgeries (Mattingly et al., 2021), and this is expected to increase as the population ages. Such increase is also found in a number of other countries. In elderly patients, postoperative neurocognitive disorders constitute one of the most common complications of surgery in older adults (Suwanabol et al., 2022; Evered et al., 2018). Meanwhile, preoperative cognitive impairment is a risk factor for not only postoperative delirium and major neurocognitive disorder but also for prolonged length of stay, readmission, and discharge to assisted care (Culley et al., 2017; Chen et al., 2022).

Preoperative cognitive screening is recommended by practice guidelines and consensus statements from the American College of Surgeons, the American Geriatrics Society, and the American Society of Anesthesiologists (Peden et al., 2021; Mohanty et al., 2016). A 1995 consensus statement on assessment of cognitive outcomes after cardiac surgery provided guidance on selection of neuropsychological tests, including recommendation of a core battery of tests, timing of assessments, and considerations during performance of the assessments (Murkin et al., 1995). However, a recent review of 274 studies published between 1995 and 2019 on postoperative cognitive dysfunction found considerable heterogeneity in testing batteries, with more than 250 unique tests used, making comparisons across studies challenging and raising the need for standardized tests that target multiple cognitive domains (Borchers et al., 2021).

The Uniform Data Set (UDS) Neuropsychological Battery has been used since 2005 by the National Institute on Aging's Alzheimer's Disease Research Centers Program (Morris et al., 2006). These standardized neuropsychological tests have been performed on over 52,000 participants, including those with normal cognition, mild cognitive impairment, and dementia.^2^ The UDS has been used to study cognitive decline associated with normal aging, Alzheimer's disease (Kiselica et al., 2024), Parkinson's disease (Lea et al., 2021), and chronic traumatic encephalopathy (Alosco et al., 2021). The UDS was revised in 2018 to the current UDS version 3. A modified UDS for remote administration, known as the tele-adapted neuropsychological battery (T-cog), has been adopted since the COVID-19 pandemic and demonstrates good reliability (Gierzynski et al., 2024; Howard et al., 2023; Loizos et al., 2021; Smith et al., 2023). Key differences are the removal of visuospatial tests, such as the Benson complex figure test, the multilingual naming test (MINT), and the drawing portions of the Montreal Cognitive Assessment (MoCA), as well as the augmentation of the trail making tests into oral versions requiring use of the same cognitive domains but without use of pen and paper. These modifications reflect a growing emphasis on flexibility and accessibility in cognitive assessment methodologies.

Use of telehealth accelerated during the COVID-19 pandemic as a safe and effective alternative to in-person visits. Advantages of telehealth visits include convenience, access, and reduced travel costs. Accordingly, the use of remote cognitive assessments has been shown to be feasible in studying perioperative neurocognitive disorders. Studies thus far have used screening tools such as the MoCA Blind/Telephone, the Ascertain Dementia Eight-item Questionnaire, the Centers for Disease Control cognitive question, and the Modified Telephone Interview for Cognitive Status in the preoperative setting (Yan et al., 2024; Cooper et al., 2022). A prospective study of patients undergoing major gynecologic oncologic surgery used the Mini-Mental State Exam (MMSE) in person and virtually, preoperatively and up to 3 months postoperatively (Makkar et al., 2024). However, more extensive remote neuropsychological testing has not been used in the perioperative population. Remote comprehensive cognitive assessment is important, however, given the high prevalence of cognitive decline associated with surgery in older adults and the expanded role of telehealth in medicine and research.

Here, we examined the feasibility of using the remotely administered T-cog for perioperative cognitive assessment in older adults scheduled for total joint lower extremity arthroplasty. We found that completion rates for the UDS v3.0 T-cog achieves near 100%. Furthermore, patients reported overall high satisfaction with the testing battery and preferred it to in-person testing. In our study, we found a rate of cognitive impairment that is comparable to prior studies of patients in this population. These results indicate that the UDS v3.0 T-cog is both a feasible and a practical tool for assessing cognitive function in the older adult perioperative population.

## 2 Materials and methods

The study protocol was approved by the New York University Grossman School of Medicine Institutional Review Board (6/27/2023, #i23-00664) and conducted in accordance with the latest revision of the Declaration of Helsinki. Written informed consent was obtained from all participants.

### 2.1 Study population

Participants were recruited from the NYU Langone Health System from November 25, 2023 to November 25, 2024. Inclusion criteria were: (1) adults aged ≥ 65 years, (2) undergoing total joint lower extremity arthroplasty, (3) American Society of Anesthesiologist (ASA) physical status I-III, and (4) willing and able to provide informed consent and participate in study procedures. The exclusion criteria included: (1) pre-existing dementia, (2) history of schizophrenia, epilepsy, craniotomy or cerebrovascular accident (stroke and/or hemorrhage), and (3) unwillingness to give informed consent. Participants who failed the UDS v3.0 T-cog hearing verification before beginning the exam were not included.

### 2.2 Study procedures and measures

#### 2.2.1 Demographic data and medical history

Data on patients' age, medical histories, and medications were extracted from Epic electronic health records (Epic Systems Corporation, Madison, WI, USA). Additional patient-reported data, including information on demographic characteristics (e.g., ethnicity, race, educational level, and gender) were collected using HIPAA-compliant online surveys administered via the Research Electronic Data Capture (REDCap) platform.

#### 2.2.2 Assessment of cognitive function

All cognitive evaluations were conducted remotely using the National Alzheimer's Coordinating Center's (NACC) tele-adapted neuropsychological test battery UDS v3.0 T-cog (form C2T, May 2020). The test battery was administered based on patient preference via telephone or through the HIPAA-compliant video conferencing platform Zoom (Zoom Video Communications Inc, San Jose, CA, USA). Assessments were audio recorded to ensure quality control of staff training and to verify patient responses. Cognitive testing was conducted by a trained study team member under the supervision of a licensed neuropsychologist.

The UDS v3.0 T-cog comprises ten tests assessing cognitive domains including global functioning, executive functioning, learning, verbal and auditory memory, attention, language, and processing speed (Table 1; Gierzynski et al., 2024). Participants were instructed to complete the assessments independently in a quiet room with minimal distractions and a stable telephone or internet connection.

**Table 1:** UDS 3.0 T-cog tests and respective domains evaluated.

**Test**	**Domain**
MoCA Blind	Global functioning, dementia severity
Craft Story 21 Recall	Learning and memory
Rey Auditory Verbal Learning Test (RAVLT)	Learning and memory
Number Span Test Forward	Attention
Number Span Test Backward	Attention
Oral Trail Making Test A	Processing speed
Oral Trail Making Test B	Executive functioning, processing speed
Category Fluency (animals, vegetables)	Language, semantic verbal fluency
Verbal Fluency Phonemic Test (F and L words)	Language, phonemic verbal fluency
Verbal Naming Test	Language, auditory verbal naming

#### 2.2.3 Assessment of participant satisfaction

Participant satisfaction was evaluated using four survey questions designed to assess the technical accessibility of the Zoom or telephone call, satisfaction with the duration of the cognitive assessment, the likelihood of participation if assessments were offered only in-person, and overall satisfaction with the remote cognitive assessment process (Table 2).

**Table 2:** Satisfaction survey.

**Question**	**Possible responses**
How technically challenging was it for you to participate in the remote cognitive assessment?	Very Easy, Easy, Neutral, A Little Challenging, Very Challenging
How satisfied were you with the length of the remote cognitive assessment?	Too Short, Just Right, Neutral/No Opinion, Too Long But Necessary, Unnecessarily Long
If the cognitive assessment had been available only *in person*, how likely would you have been to participate in the study?	Very Likely, Likely, Neutral, Unlikely, Very Unlikely
How would you rate your overall experience with the remote cognitive assessment?	Very Satisfied, Satisfied, Unsatisfied, Very Unsatisfied

#### 2.2.4 Outcome measures

##### 2.2.4.1 Pre-existing cognitive impairment

Pre-existing cognitive impairment was assessed through the MoCA Blind component of the UDS v3.0 T-cog test battery. Participants could score a maximum of 22 points. A score adjusted for education and lower than 18 points was used to indicate the presence of cognitive impairment (Wittich et al., 2010). Z-scores for most tests were calculated using a regression-based, normative score calculator (Weintraub et al., 2018; Shirk et al., 2011). The RAVLT Z-scores were derived from the Mayo's Normative Studies Scoring Resource, which used data from the Mayo Older Americans Normative Studies (MOANS; Stricker et al., 2021). Oral Trail Making test scores were calculated using normative data from Mrazik et al. (2010), as recommended by the NACC.

##### 2.2.4.2 Cognitive assessment completion rates

A cognitive assessment was defined as completed once the full UDS v3.0 T-cog test battery was completed. Any interruption in completion was noted down and the assessment was marked as incomplete.

##### 2.2.4.3 Validity checklist for participant's responses

The UDS v3.0 T-cog provides a validity checklist for researchers to indicate whether environmental distractions, interruptions, lack of patient engagement or effort, hearing difficulties, or other factors significantly influenced test results. Options of validity included “Very valid, probably accurate indication of participant's cognitive abilities,” “Questionably valid, possibly inaccurate indication of participant's cognitive abilities,” and “Invalid, probably inaccurate indication of participant's cognitive abilities” for the researchers to select.

#### 2.2.5 Statistical analysis

Results were expressed as a number (percentage) for categorical variables and mean (± standard deviation) for continuous variables. Hypothesis testing was performed using the chi-square test for binary variables and the non-parametric Mann-Whitney *U*-test for continuous variables comparing means between two or multiple groups. *P* < 0.05 was considered significant and all tests were two-tailed. All statistical analyses were performed using Microsoft Excel version 16.91 (Microsoft Corporation, Redmond, WA, USA) and IBM SPSS 30 (IBM Corp. Armonk, NY, USA).

## 3 Results

### 3.1 Baseline characteristics of the study cohort of patients prior to undergoing lower extremity joint replacement surgery

A summary of the baseline characteristics of the study population is presented in Table 3. The study cohort consisted of 49 female participants (60%) and 32 male participants (40%). The sample was predominantly white (*n* = 62; 77%), followed by Black/African American (*n* = 10; 12%) and Asian (*n* = 4; 4.9%). Of the total, 60 participants (74%) identified as non-Hispanic/Latino, while nine participants (11%) identified as Hispanic/Latino. The mean age of participants was 74 years (± 5.2). Fifteen participants (19%) were using opioids for chronic pain, nine participants (11%) were using gabapentinoids, and three participants (3.7%) were using benzodiazepines.

**Table 3:** Demographics and characteristics of the study population.

	**Pre-operative cognitive impairment** ^**a**^			
**Demographics**	**No**	**Yes**	**Total**	***p*** **-value**
	**(*****n*** = **58)**	**(*****n*** = **23)**	**(*****n*** = **81)**	
**Gender (%)**				0.335
Male	21 (36)	11 (48)	32 (40)	
Female	37 (64)	12 (52)	49 (60)	
**Age, mean (±SD)**	73.6 ± 5.0	75.4 ± 5.6	74.1 ± 5.2	0.389
**Age group, range (%)**				0.236
65–69	14 (24)	4 (17)	18 (22)	
70–74	15 (26)	5 (22)	20 (25)	
75–79	22 (38)	10 (44)	32 (40)	
80–84	6 (10)	1 (4.0)	7 (8.6)	
85+	1 (2.0)	3 (13)	4 (4.9)	
**Years of education, mean (±SD)** ^**b**^	17.9 ± 2.6	16.5 ± 3.2	17.5 ± 2.8	0.070
**Level of education**				0.102
High School Diploma	3 (5.0)	4 (17)	7 (8.6)	
College Degree	15 (26)	3 (13)	18 (22)	
Associate Degree	5 (8.5)	3 (13)	8 (9.9)	
Bachelor's Degree	0 (0.0)	0 (0.0)	0 (0.0)	
Master's Degree	0 (0.0)	0 (0.0)	0 (0.0)	
Doctoral Degree	31 (54)	7 (30)	38 (47)	
Missing Data	4 (7.0)	6 (26)	10 (12)	
**Race (%)**				0.189
White	47 (81)	15 (65)	62 (77)	
Black/African American	7 (12)	3 (13)	10 (12)	
Asian	1 (2.0)	3 (13)	4 (4.9)	
American Indian/Alaskan American	1 (2.0)	0 (0.0)	1 (1.2)	
Unknown^c^	2 (3.5)	2 (9.0)	4 (4.9)	
**Ethnicity (%)**				0.073
Hispanic or Latino	5 (8/5)	4 (17)	9 (11)	
Not Hispanic or Latino	46 (79)	14 (61)	60 (74)	
Not Reported/Unknown^c^	7 (12)	5 (22)	12 (15)	
**Past medical history, yes (%)**				
Hypertension	37 (64)	17 (74)	54 (67)	0.384
Hypercholesterolemia and/or hyperlipidemia	36 (62)	11 (52)	47 (58)	0.242
Obesity	20 (35)	7 (30)	27 (33)	0.727
History of Malignancy (Active or Past)	16 (28)	7 (30)	23 (28)	0.798
Thyroid Disease	13 (22)	5 (22)	18 (22)	0.947
Psychiatric Disorder (Anxiety and/or depression)	12 (21)	6 (26)	18 (22)	0.598
Lung Disease (Asthma and/or COPD)	11 (19)	6 (26)	17 (21)	0.478
Coronary Artery Disease	10 (17)	5 (22)	15 (19)	0.638
Diabetes	9 (16)	4 (17)	13 (16)	0.836
Sleep Apnea	7 (12)	5 (22)	12 (15)	0.269
**Smoking history (current or former), yes (%)**	24 (41)	12 (52)	36 (44)	0.378
**History of alcohol overconsumption, yes (%)**	9 (16)	1 (4.0)	10 (12)	0.168
**Medication history (%)**				
Opioids	11 (19)	4 (17)	15 (19)	0.869
Anti-inflammatory agents (NSAIDs)	22 (38)	10 (44)	32 (40)	0.645
Gabapentinoids	4 (7.0)	5 (22)	9 (11)	0.055
Benzodiazepines	3 (5.0)	0 (0.0)	3 (3.7)	0.266
Antidepressants	8 (14)	4 (17)	12 (15)	0.681
**Type of surgery (%)**				0.293
Knee Arthroplasty	33 (57)	16 (70)	49 (60)	
Hip Arthroplasty	25 (43)	7 (30)	32 (40)	

^a^Cognitive impairment was determined based on the MOCA Blind test, with normal scores being ≥18/22 and where those scoring < 18 would be considered to have abnormal scores and thus, cognitive impairment (without determined severity degree).

^b^Years of education was calculated as the summarized number of years according to previously plotted neuropsychiatric data, with finished: high school = 12 years, bachelors = 4 years, masters = 2 years, doctoral = 4 years, associate degree = 2 years (only if completed).

^c^Patients preferred not to respond.

### 3.2 Completion rates for the UDS v3.0 T-cog battery among patients prior to undergoing lower extremity joint replacement surgery

Out of the 81 patients enrolled, 80 participants completed the full UDS v3.0 T-cog assessment (99% completion rate). The UDS v3.0 T-cog allows the optional administration of the RAVLT Delayed and Recognition tests, and four subjects did not complete these tests due to lack of time. One participant did not finish the full assessment due to fatigue but completed four out of 10 tests; however, the researcher's perceived validity of their completed tests was still captured.

### 3.3 Researcher's perceived validity of the T-cog battery

**Figure 1:**
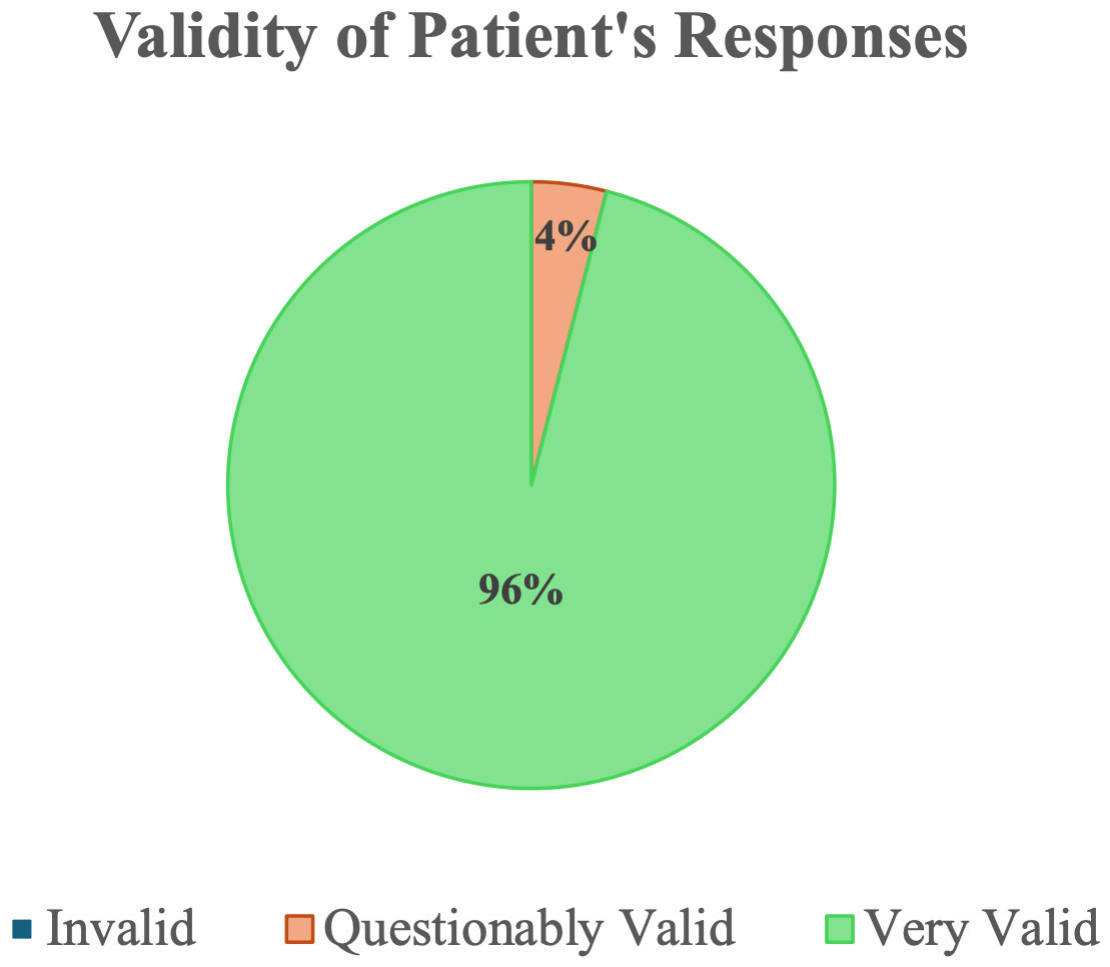
Validity of patient responses as assessed by proctors in the UDS v3.0 (Form 2CT).

Each validity assessment was evaluated directly after the conclusion of the UDS v3.0 T-cog by the same researcher administering the cognitive assessment. As presented in Figure 1, 96% of the cognitive assessments were rated as a “Very valid” representation of the subject's cognitive abilities by the research coordinator administering the test via the UDS v3.0 T-cog Validity Checklist provided by the NACC, while 4% were classified as “Questionably valid.” None were categorized as “Invalid.” The three assessments scored as “Questionably valid” assessments were administered via telephone, while 100% of video assessments were scored as “Very valid.” Distractions were identified as the primary reason for questionable validity.

One researcher conducted 37 assessments, of which 30 were via video and seven were via telephone. One hundred percent of video assessments were rated as “Very valid” and two telephone assessments were rated as “Questionably valid.” Another researcher conducted a total of 21 assessments, of which eight were via video and 13 were via telephone; all video assessments were rated as “Very valid,” and one telephone assessment was rated as “Questionably valid.” Another researcher conducted 15 assessments, of which 13 were video assessments and two were phone assessments, all scored as “Very valid.” Two other researchers each conducted four video assessments, and all of their assessments were scored as “Very valid.”

### 3.4 Pre-existing cognitive impairment in older patients prior to joint replacement surgery assessed by MoCA blind cut-off scores

**Figure 2:**
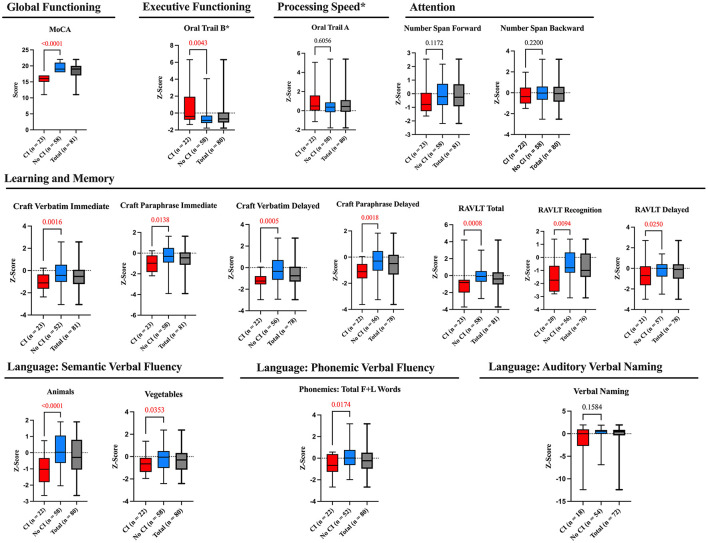
Result cognitive assessments according to cognitive domain. Comparison of *Z*-scores between participants with CI (using MoCA cut-off scores) vs. those without. Box plots present the means (with min-max) for each test battery according to the cognitive domain assessed and the non-parametric Mann-Whitney *U*-test was applied to compare means between the two groups. Negative *Z*-scores indicate worse performance compared to normative data, except for the Oral Trails tests, where a positive score indicates a longer time to achieve the task, and therefore worse performance. *Oral Trail B also assesses processing speed.

Out of the 81 participants who completed the MoCA Blind, 23 participants scored lower than 18, classifying them as having pre-existing cognitive impairment (28.4%), where age, years of education and gender did not differ among those with vs. without cognitive impairment using univariate comparisons between groups (*p* = 0.389, *p* = 0.070, and *p* = 0.335, respectively; Wittich et al., 2010). Scores were statistically significantly different between participants with cognitive impairment (CI) vs. those without (*p* < 0.0001, see Figure 2).

### 3.5 Patients with cognitive impairment demonstrate impairment across multiple cognitive tests

Box plots showing *Z*-score means for all separate tests classified according to the cognitive domains assessed are shown in Figure 2. Negative *Z*-scores indicate worse performance compared to normative data, except for the Oral Trails tests, where a positive score indicates a longer time to achieve the task, and therefore worse performance. When comparing *Z*-scores between the two groups (CI vs. no-CI), there was a significant difference in *Z*-scores for all tests (*p* < 0.05) except those assessing attention (number span tests) and auditory verbal naming.

### 3.6 Patient satisfaction with the T-cog

**Figure 3:**
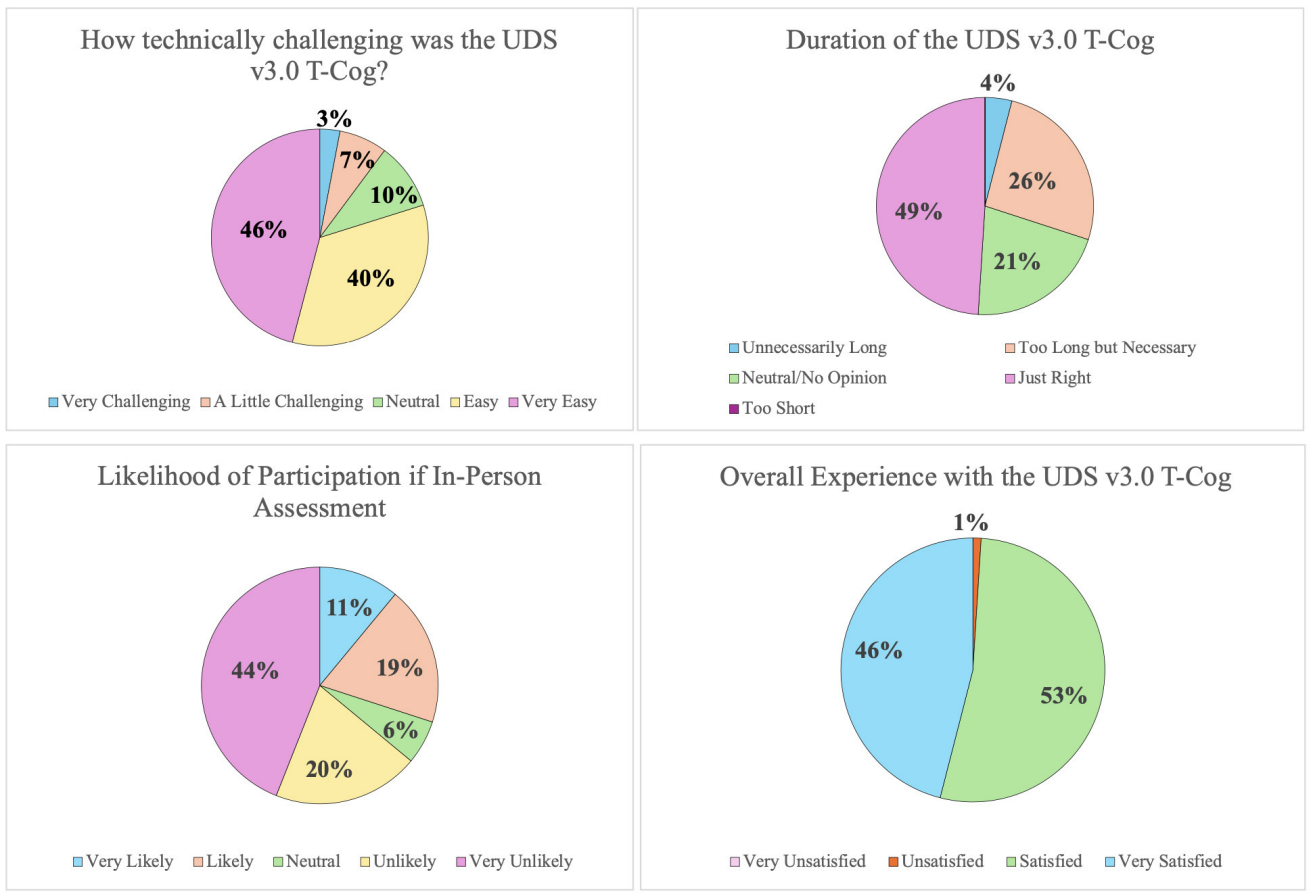
Results of feasibility and participant satisfaction survey.

Out of 81 participants, a total of 70 participants completed the satisfaction survey (86%). As presented in Figure 3, the majority of participants found the UDS v3.0 T-cog easy to use, just right in length, and would be unlikely to participate in the study if the assessment were done in-person. The overall satisfaction with the UDS v3.0 T-cog was high, with 99% being satisfied or very satisfied.

## 4 Discussion

In this study, we tested the feasibility of using a remotely conducted UDS v3.0 T-cog to assess cognitive function in a unique population of older adults who are about to undergo lower extremity surgery. We found that the UDS v3.0 T-cog is user-friendly, allows near total completion rates, and is feasible to administer before surgery to older adults. Further, we found that ~28% of the patients in our cohort showed signs of cognitive impairment.

To the best of our knowledge, this is the first study to utilize a comprehensive UDS v3.0 cognitive testing battery to study cognitive function in older patients in a perioperative setting. In the last 5 years, remote assessment of cognitive function has gained considerable traction (Katz et al., 2021; Chappelle et al., 2023; Lai et al., 2022), and UDS v3.0 T-cog has been advocated by the National Institute on Aging's Alzheimer's Disease Research Centers Program, been widely adopted (Sachs et al., 2024; Hackett et al., 2021), and shown validity (Gierzynski et al., 2024; Howard et al., 2023; Smith et al., 2023). In our study, we are approaching a patient population who are more difficult to enroll for in-person assessment, due to their impending surgery. There is increased interest, however, to characterize cognitive function in this patient population. Screening tests have been used for remote preoperative cognitive assessments (Yan et al., 2024; Cooper et al., 2022; Yu et al., 2021), however, few studies have focused on using comprehensive assessment tools in this patient population. This is important, as older adults may manifest cognitive deficits in different domains (Cullum et al., 2000). Surgery and pain may affect different dimensions of cognition, and a comprehensive battery allows assessment of which cognitive dimension(s) may be affected. Likewise, certain treatments may affect one or more dimensions of cognition selectively. Further, more detailed assessment allows more detailed studies on mechanisms of cognitive decline, especially as it allows associations of behavioral outcomes with genetic/genomic and neurophysiological data. Our study shows that overall completion rate for remote assessment is very high. Interestingly, not only do patients in our cohort demonstrate overall satisfaction with the remote UDS v3.0 T-cog, but they also endorse preference for the remote assessment to in-person assessment. In fact, our data completion rate compares favorably to in-person studies, which typically demonstrate 78–95% data completion rate (Ballard et al., 2012; Moller et al., 1998; Silbert et al., 2015; Berger et al., 2022).

In our study, we found that 28% of patients showed cognitive impairment (CI), a finding consistent with previously reported data in the same age group with other remotely conducted or in-person conducted studies (Culley et al., 2016; Kapoor et al., 2022; Susano et al., 2020). Additionally, participants with CI demonstrated significantly lower performance across multiple cognitive domains, particularly those related to learning and memory (Figure 2). These results further support the validity and feasibility of the UDS v3.0 T-cog as a reliable screening tool for cognitive impairment.

The feasibility of and patient satisfaction with the UDS v3.0 T-cog assessment is promising for longitudinal testing. Indications for longitudinal assessment are multiple. First, longitudinal assessment can be performed for patients at risk for developing dementia (Cruz-Almeida et al., 2019). Second, it allows us to correlate behavioral findings with blood or cerebrospinal fluid biomarkers. Third, longitudinal use of UDS v3.0 T-cog can be performed to assess potential adverse effects on cognition for a number of drugs. Finally, it can be used to study progression or emergence of cognitive decline after trauma or surgery, such as for studies of postoperative cognitive function.

While our study has demonstrated overall feasibility for the remote UDS, there are some limitations. We have used two different options for remote assessment—an audio only option and a video option. A video option allows observation of patients and potentially improved ability of researchers to assess perceived validity with the T-cog Validity Checklist. However, audio allows patient participation in cases in which full video-conferencing capability is lacking. In our study, 73% utilized video assessments over the telephone option. Furthermore, this is a single center study, and thus future multi-center studies are needed to further demonstrate feasibility, especially for populations where access to remote testing is more limited. Another limitation of remote assessment is that it may not fully capture all facets of executive function such as visuospatial ability. Lastly, Verbal Naming Test *Z*-scoring can be confounded by patients who learned English as a second language, as these patients may have scored poorly on this task due to translation challenges rather than cognitive impairment.

## 5 Conclusion

In this study we have shown the feasibility of a remotely conducted UDS neuropsychological testing battery in a perioperative patient population. We found that ~28% of patients over the age of 65 in this clinical setting showed cognitive impairment. Importantly, the UDS v3.0 T-cog is well-tolerated and feasible to administer in the real-world setting. These results support the use of the UDS v3.0 T-cog to assess cognitive function of patients in the perioperative setting.

## Data Availability

The raw data supporting the conclusions of this article will be made available by the authors upon reasonable request.

## Footnotes

^1^ https://www.census.gov/data/tables/2023/demo/popproj/2023-summary-tables.html

^2^ https://naccdata.org/
